# Systematic Analysis of the Crystal Chemistry and Eu^3+^ Spectroscopy along the Series of Double Perovskites Ca_2_LnSbO_6_ (Ln = La, Eu, Gd, Lu, and Y)

**DOI:** 10.1021/acs.inorgchem.1c00932

**Published:** 2021-05-21

**Authors:** Fabio Piccinelli, Irene Carrasco, Chong-Geng Ma, Marco Bettinelli

**Affiliations:** † Luminescent Materials Laboratory, Department of Biotechnology, Università di Verona and INSTM, UdR Verona, Strada Le Grazie 15, Verona 37134, Italy; ‡ Département Microélectronique & Microcapteurs, Université de Rennes, CNRS, ISCR−UMR 6226, ScanMAT−UMS 2001, IETR−UMR 6164, Rennes F-35000, France; § CQUPT-BUL Innovation Institute, Chongqing University of Posts and Telecommunications, Chongqing 400065, P.R. China

## Abstract

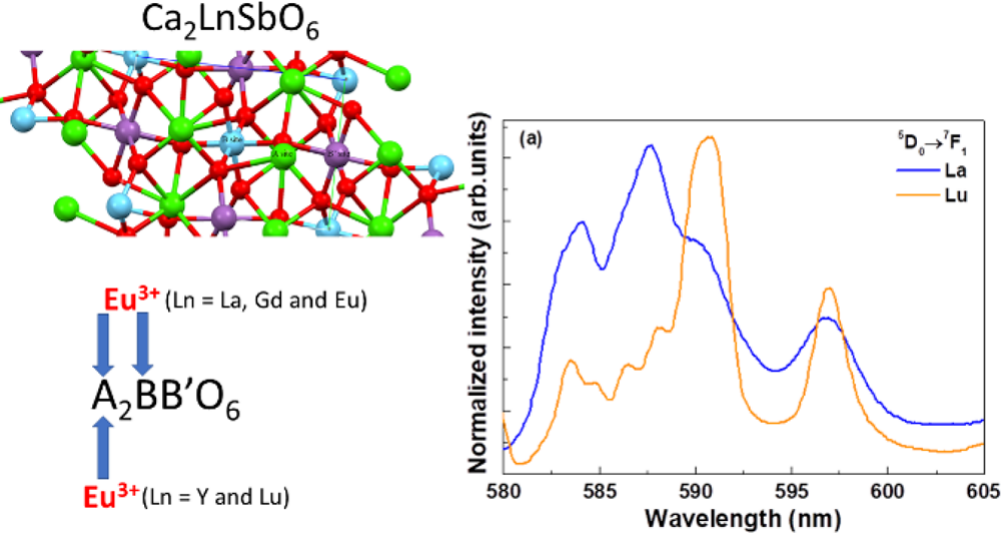

Eu^3+^ (1 mol %)-doped Ca_2_LnSbO_6_ (replacing Ln^3+^; Ln = Lu, Y, Gd, and La) and Ca_2_EuSbO_6_ were synthesized and structurally characterized by means of X-ray powder diffraction. The Eu^3+^ luminescence spectroscopy of the doped samples and of Ca_2_EuSbO_6_ has been carefully investigated upon collection of the excitation/emission spectra and luminescence decay curves of the main excited states. Surprisingly, apart from the dominant red emission from ^5^D_0_, all the doped samples show an uncommon blue and green emission contribution from ^5^D*_J_* (*J* = 1, 2, and 3). This is made possible thanks to both multiphonon and cross-relaxation mechanism inefficiencies. However, the emission from ^5^D_3_ is more efficient and the decay kinetics of the ^5^D*_J_* (*J* = 0, 1, and 2) levels is slower in the case of Y- and Lu-based doped samples. This evidence can find a possible explanation in the crystal chemistry of this family of double perovskites: our structural investigation suggests an uneven distribution of the Eu^3+^ dopant ions in Ca_2_YSbO_6_ and Ca_2_LuSbO_6_ hosts of the general A_2_BB′O_6_ formula. The luminescent center is mainly located in the A crystal site, and on average, the Eu–Eu distances are longer than in the case of the Gd- and La-based matrix. These longer distances can further reduce the efficiency of the cross-relaxation mechanism and, consequently, the radiative transitions are more efficient. The slower depopulation of Eu^3+ 5^D_2_ and ^5^D_1_ levels in Ca_2_YSbO_6_ and Ca_2_LuSbO_6_ hosts is reflected in the longer rise observed in the ^5^D_1_ and ^5^D_0_ decay curves, respectively. Finally, in Ca_2_EuSbO_6_, the high Eu^3+^ concentration gives rise to an efficient cross-relaxation within the subset of the lanthanide ions so that no emission from ^5^D*_J_* (*J* = 1, 2, and 3) is possible and the ^5^D_0_ decay kinetics is faster than for the doped samples.

## Abstract 2

Eu^3+^ as a dopant ion in the series of double perovskite Ca_2_LnSbO_6_ (Ln = La, Gd, Y, and Lu) hosts shows a different occupation of the available A and B crystal sites; this strongly affects the luminescence behavior in the visible spectral region.

## 1 Introduction

Rare earth double perovskite materials with the general formula A_2_BB′O_6_ are characterized by interesting magnetic and dielectric properties.^1−3^ The main structural motif of these compounds consists of a network of alternating BO_6_ and B′O_6_ octahedra, with A-atoms occupying the 12-coordinated interstitial spaces between octahedra. Depending on the nature and size of the other elements, the rare earth ions can occupy the A-site or B-site. In the Ca_2_LnRuO_6_ (Ln = La–Lu) system,^4^ which crystallizes in the monoclinic *P*2_1_/*n* space group, the Ca^2+^ and Ln^3+^ cations are partially disordered in the A-site and B-site positions of the A_2_BB′O_6_ double perovskite, and the Ru(V) cations are located at the B′-site; therefore, the general formula of these compounds is (Ca_2–*x*_Ln*_x_*)(Ln_1–*x*_Ca*_x_*)RuO_6_. The abundance of Ln^3+^ located at the B-site varies with its cationic radius: the larger Ln cations tend to occupy the A-site, whereas the smaller Ln cations tend to enter the B-site. Similar crystal chemistry is expected for antimonates with double perovskite materials and Ca_2_LnSbO_6_ formula. Although a systematic study on their crystal chemistry is still missing in the literature, two components of this family (Ca_2_LaSbO_6_ and Ca_2_YSbO_6_) have been effectively employed as hosts of luminescent trivalent lanthanide ions. In particular, Ca_2_LaSbO_6_, which can be obtained with Eu^3+^ up to 80% substituting La^3+^, has been considered a useful red phosphor.^5,6^ Another efficient red phosphor can be obtained by doping Ca_2_YSbO_6_ with Eu^3+^ ion. The codoping with Bi^3+^ has been reported to enhance the intensity of the red emission.^7^ Considering the crystal chemistry of the host, Y^3+^ ions are supposed to occupy only the centrosymmetric B site. Nevertheless, Eu^3+^ should be located in a noncentrosymmetric crystal site, since the ^5^D_0_ → ^7^F_2_ band dominates the luminescence spectrum.^8^ Since Ca^2+^ is located in the A-site (*C*_1_ point symmetry), the authors reasonably assumed a Ca^2+^/Eu^3+^ substitution and the presence of a charge compensation mechanism. Finally, Ca_2_YSbO_6_ is also an effective host for other luminescent ions, such as trivalent Sm, Dy, Ho, and Er.^9^

Due to the lack of a comprehensive study on the crystal chemistry of the Ca_2_LnSbO_6_ family, we have found it interesting to undertake a structural study on Ca_2_LaSbO_6_, Ca_2_GdSbO_6_, Ca_2_LuSbO_6_, and Ca_2_YSbO_6_ doped with 1 mol % Eu^3+^, and neat Ca_2_EuSbO_6_, by means of X-ray diffraction. The effects of the different nature of the hosts on Eu^3+^ luminescence spectroscopy have been also discussed and some structural details have been revisited. This study, focusing on the structural/spectroscopic relationship, reveals the presence of unusual spectroscopic features of Eu^3+^ when introduced as an impurity in these antimonate hosts.

## 2 Experimental Section

### 2.1 Materials and Synthesis

Crystalline samples of 1 mol % Eu^3+^-doped Ca_2_LnSbO_6_ (replacing Ln^3+^; Ln = Lu, Y, Gd, and La) and Ca_2_EuSbO_6_ were prepared by solid-state reaction in and air atmosphere. CaCO_3_ (>99%), Sb_2_O_5_ (99.995%), Ln_2_O_3_ (Ln = Y and La, 99.99%; Ln = Lu and Gd, 99.9%), and Eu_2_O_3_ (99.99%) were thoroughly mixed and pressed into pellets under a pressure of 10 tons. The samples underwent two heat treatments: the first one at 600 °C for 6 h to eliminate carbonates and the second one at 1400 °C for 24 h with a slow cooldown of 3 °C/min. Intermediate grindings were performed to improve the homogeneity of the materials.

### 2.2 Structural Investigation

X-ray diffraction (XRD) patterns were measured with a Thermo ARL X’TRA powder diffractometer, operating in the Bragg–Brentano geometry and equipped with a Cu-anode X-ray source (Kα, λ =1.5418 Å), using a Peltier Si(Li)-cooled solid-state detector. The patterns were collected with a scan rate of 0.002°/s in the 18–120° 2θ range. Polycrystalline antimonate samples were ground in a mortar and then put in a side-loading sample holder for data collection.

The General Structure Analysis System (GSAS) program was employed for the Rietveld refinement calculations.^10^ The instrumental X-ray peak profile functions and the sample displacement (SHFT variable) were determined by Rietveld refinement of the diffraction pattern of the LaB_6_ powder standard reference material (NIST 660C).

The reference structural model exploited in the Rietveld calculation was the one pertaining to the isostructural perovskite-like Ca_3_TeO_6_ determined in a study by Hottentot and Loopstra^11^ in which Sb has been located in the place of Te and Ca, and Ln ions shared the two crystallographic positions of Ca._._ The following structural refinement strategy has been performed: (i) refinement of the background functions (shifted Chebyschev), scale factor, and cell parameters; (ii) refinement of the occupation factors (OFs) of Ca and Ln in the two available crystal sites; (iii) refinement of the fractional atomic coordinates for Ca/Ln in the 4*e* crystal site (site A); (iv) refinement of the fractional atomic coordinates for the oxygen atoms; (v) refinement of the isotropic thermal parameter (*U*_iso_) for Ca, Ln, and Sb ions; (vi) refinement of the isotropic thermal parameter (*U*_iso_) for oxygen atoms (we do not observe significant improvement of the refinement employing anisotropic thermal parameters); (vii) global refinement of all structural variables mentioned above. In each step of the Rietveld calculation, also the GU, GV, GW, LX, LY, and asym profile terms of the pseudo-Voigt profile function no. 2, included in the GSAS program, were refined. Due to its very low concentration (1 mol %), the presence or absence of Eu^3+^ within the structural model is expected to not change, in a significant way, the result of the Rietveld refinements. For this reason, for the sake of simplicity, the presence of Eu^3+^ is not considered during the structural calculation. Crystal data such as atomic fractional coordinates, OFs, and *U*_iso_ for 1% Eu^3+^-doped Ca_2_LnSbO_6_ and Ca_2_EuSbO_6_ are reported in the Supporting Information (Tables S1–S6), along with other relevant powder diffraction data (see the Powder Diffraction Data section).

### 2.3 Spectroscopic Investigation

Room-temperature luminescence spectra and decay curves were measured with a Fluorolog 3 (Horiba-Jobin Yvon) spectrofluorometer, equipped with a Xe lamp, a double excitation monochromator, a single emission monochromator (mod. HR320), and a photomultiplier in photon counting mode for the detection of the emitted signal. All the spectra were corrected for the spectral response of the setup.

## 3 Results and Discussion

### 3.1 Structural Determination of 1% Eu^3+^-Doped Ca_2_LnSbO_6_ (Ln = Lu, Y, Gd, and La) and Ca_2_EuSbO_6_

**Figure 1:**
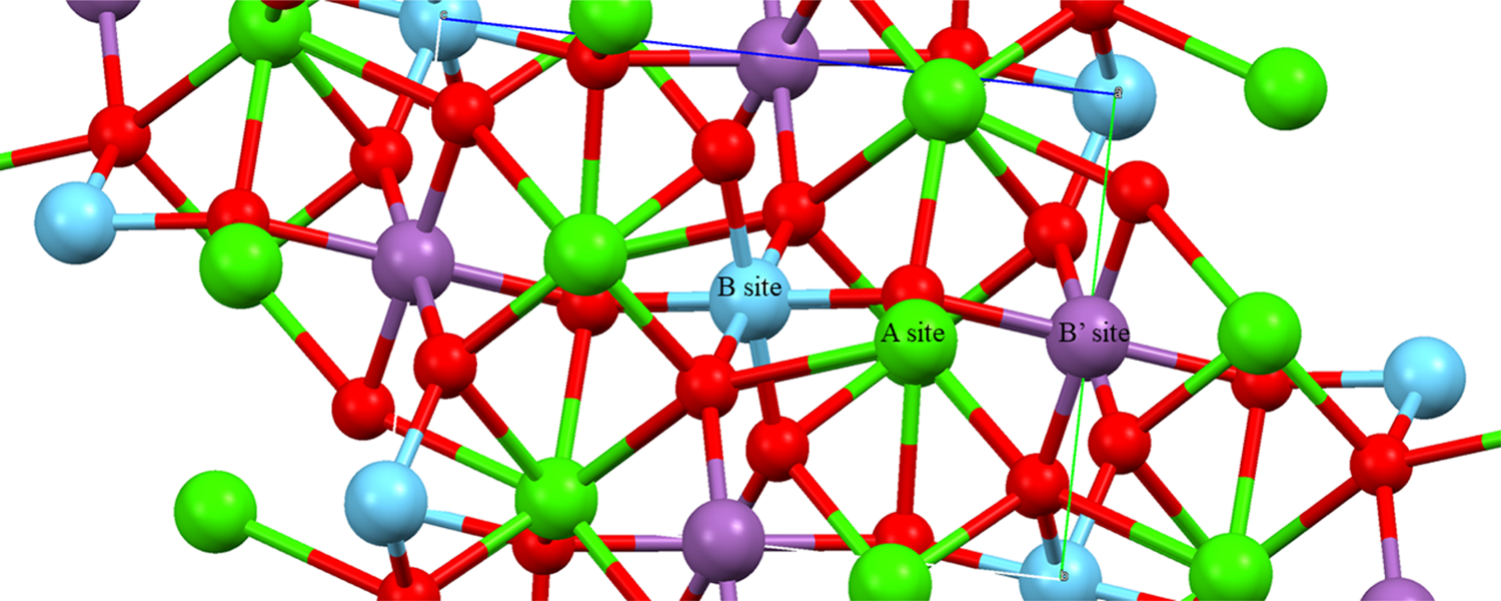
Picture of the crystal structure, along the *a* axis, of the double perovskite of the general formula A_2_BB′O_6_. The location of the cationic sites A, B, and B′ is shown. Red spheres represent oxygen atoms.

In Figure 1, the picture of the crystal structure of the investigated double perovskite is shown.

**Figure 2:**
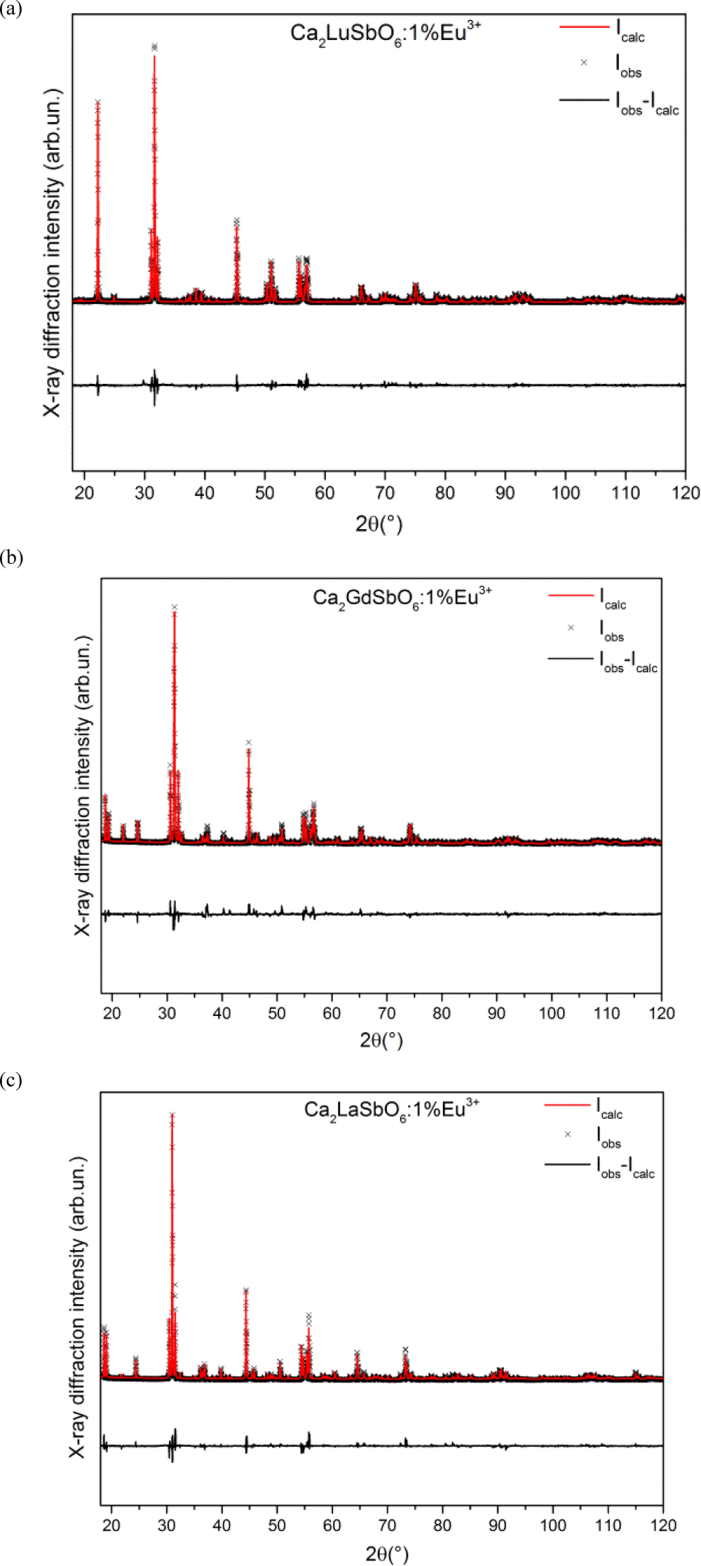
Observed (crosses) and refined (continuous red line) powder patterns of (a) 1% Eu^3+^-doped Ca_2_LuSbO_6_, (b) 1% Eu^3+^-doped Ca_2_GdSbO_6_, and (c) 1% Eu^3+^-doped Ca_2_LaSbO_6_. The observed–refined curves are shown at the bottom of each plot. The same plots for 1% Eu^3+^-doped Ca_2_YSbO_6_ and Ca_2_EuSbO_6_ are reported in Figure S1.

The observed and fitted XRD patterns of doped Ca_2_LnSbO_6_ (Ln = Lu, Gd, and La chosen as representative samples) are shown in Figure 2.

Inspection of this figure shows that there is good agreement between the observed and refined powder patterns. The refined lattice parameters and OFs are given in Tables 1 and 2, respectively, and some selected bond distances are listed in Table 3.

**Table 1:** Refined Lattice Parameters for the 1% Eu^3+^-Doped Ca_2_LnSbO_6_ Family (Ln = Lu, Y, Gd, and La) and for Ca_2_EuSbO_6_

	cell parameters (Å, °, Å^3^)				
Ln in Ca_2_LnSbO_6_ host	*a*	*b*	*c*	β	*V*
Lu	5.5711(1)	5.7530(1)	7.9958(3)	89.913(2)	253.27(2)
Y	5.5888(1)	5.8021(1)	8.0494(3)	89.970(4)	261.01(1)
Gd	5.5884(2)	5.8466(3)	8.0817(1)	89.753(5)	264.06(3)
Eu	5.5947(2)	5.8522(2)	8.0887(2)	90.255(3)	264.83(2)
La	5.6830(1)	5.8795(1)	8.1707(1)	89.913(4)	273.01(2)

**Table 2:** Occupation Factors of Ln^3+^ and Ca^2+^ in the Different Crystal Sites of the Ca_2_LnSbO_6_ Host

		OF	ionic radius (Å)^a^	
host	cation	site A (CN 8)/site B (CN 6)	CN 6	CN 8
Ca_2_LuSbO_6_	Lu^3+^	0.032(2)/0.936(3)	0.85	0.97
	Ca^2+^	0.968(2)/0.064(3)	1.00	1.12
Ca_2_YSbO6	Y^3+^	0.130(2)/0.872(2)	0.892	1.015
	Ca^2+^	0.870(2)/0.128(2)	1.00	1.12
Ca_2_GdSbO_6_	Gd^3+^	0.375(6)/0.250(5)	0.94	1.06
	Ca^2+^	0.625(6)/0.750(5)	1.00	1.12
Ca_2_EuSbO_6_	Eu^3+^	0.414(3)/0.171(2)	0.95	1.07
	Ca^2+^	0.586(3)/0.829(2)	1.00	1.12
Ca_2_LaSbO_6_	La^3+^	0.490(2)/0.020(2)	1.06	1.18
	Ca^2+^	0.510(2)/0.980(2)	1.00	1.12

a Data taken from ref (12).

**Table 3:** Average M–O Bond Distances along the Ca_2_LnSbO_6_ Host Family

	average bond distance (Å)		
Ln in Ca_2_LnSbO_6_ host	Sb–O	Ca/Ln(1)–O (site B; CN 6)	Ca/Ln(2)–O (site A; CN 8)
Lu	2.03(1)	2.18(1)	2.54(2)
Y	1.96(1)	2.28(1)	2.56(2)
Gd	1.99(1)	2.33(2)	2.55(3)
Eu	2.00(1)	2.33(2)	2.55(2)
La	2.01(1)	2.34(1)	2.61(2)

As expected, according to Vegard’s law, the cell size increases as the ionic radius of the trivalent lanthanide ions increases (Table 1). Ca^2+^ and Ln^3+^ cations are partially disordered in the A-site (*C*_1_ point symmetry) and B-site (*C_i_* point symmetry) positions of the A_2_BB′O6 double perovskite and the Ca/Ln distribution over these two available crystal sites is strongly dependent on the nature of the Ln ion. When small Lu and Y are considered, the trivalent ion shows a strong preference for the site with coordination number (CN) 6 (Table 2, site B). In the cases of Gd and Eu, the Ca/Ln distribution is almost homogeneous over the two crystal sites. Finally, the La^3+^ ion prefers to occupy the crystal site with CN = 8, while site B (CN = 6) is almost fully occupied by Ca^2+^ (Table 2). Obviously, the degree of Ca/Ln disorder is mainly dependent on the difference between their ionic radii. The bigger the difference is, the smaller the disorder is. As discussed before, this is particularly true for Ln ions smaller than Ca^2+^, e.g., the Lu ion is mainly located in the crystal site B. Moreover, it is interesting to note that on average, the Sb–O distances (around 2 Å) are not significantly affected by the nature of the trivalent ion and the Ca/Ln–O distances deviate from 2.55 Å only for Ln = La (Table 3). On the other hand, in the case of the crystal site B (CN = 6), the average Ca/Ln–O distance is around 2.33 Å for Ln = Gd, Eu, and La, while it is shorter for Y (2.28 Å) and, in particular, for Lu (2.18 Å) (Table 3 and Figure S2). This behavior is quite similar to the one observed for the Ca_2_LnRuO_6_ (Ln = La–Lu) double perovskite family, already discussed above.^4^

We also point out some discrepancy with the crystal data present in the current literature. In the case of the Ca_2_LaSbO_6_ host, while the La ion has been calculated to exclusively occupy site A, by Yin et al.,^6^ we found the presence of a small percentage of La^3+^ (2%) in the crystal site B. The most important discrepancy concerns the Ca_2_YSbO_6_ host. In the literature, Y^3+^ ions are supposed to occupy only the centrosymmetric octahedral B site,^7^ while we detect the presence of this ion also in site A (OF = 0.130, Table 2). Taking into account the substitution of Ca^2+^/Ln^3+^ ions in the crystal lattice by the luminescent Eu^3+^ (see the discussion below), this finding should have a significant impact. In fact, according to our conclusions, we can assume that Eu^3+^ could occupy both crystal sites A and B without the necessity of involving charge compensation mechanisms.

### 3.2 Luminescence of Ca_2_EuSbO_6_ and 1% Eu^3+^-Doped Ca_2_LuSbO_6_, Ca_2_YSbO_6_, Ca_2_GdSbO_6_, and Ca_2_LaSbO_6_

**Figure 3:**
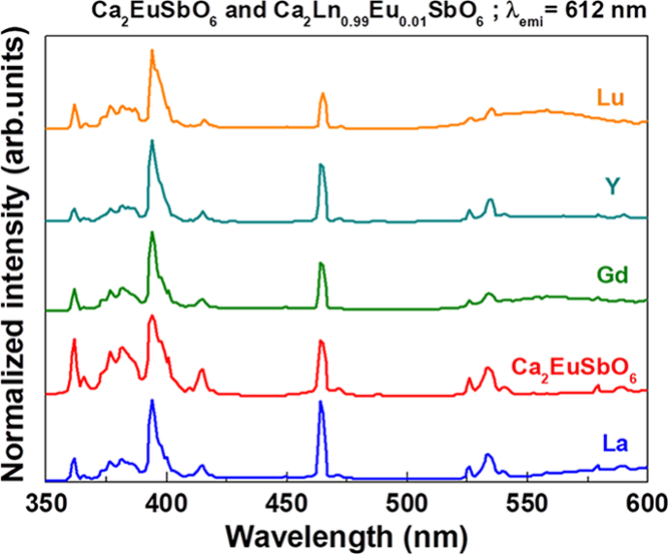
Room-temperature luminescence excitation spectra of 1% Eu^3+^-doped Ca_2_LnSbO_6_ (Ln = Lu, Y, Gd, and La) and Ca_2_EuSbO_6_.

The normalized RT excitation spectra of 1% Eu^3+^-doped Ca_2_LnSbO_6_ (Ln = Lu, Y, Gd, and La) and Ca_2_EuSbO_6_ are shown in Figure 3. The spectra have been normalized to the *I*_max_ of the ^7^F_0_ → ^5^L_6_ transition.

All compounds show various intense Eu^3+^ excitation peaks [around 362 nm (^7^F_0_ → ^5^D_4_ transition); 394 nm (^7^F_0_ → ^5^L_6_ transition); 415 nm (^7^F_0_ → ^5^D_3_ transition); 464 nm (^7^F_0_ → ^5^D_2_ transition); 526 nm (^7^F_0_ → ^5^D_1_ transition); 534 nm (^7^F_1_ → ^5^D_1_ transition)]. Also, an O → Eu charge transfer (CT) band below 300 nm is detected (not shown).

**Figure 4:**
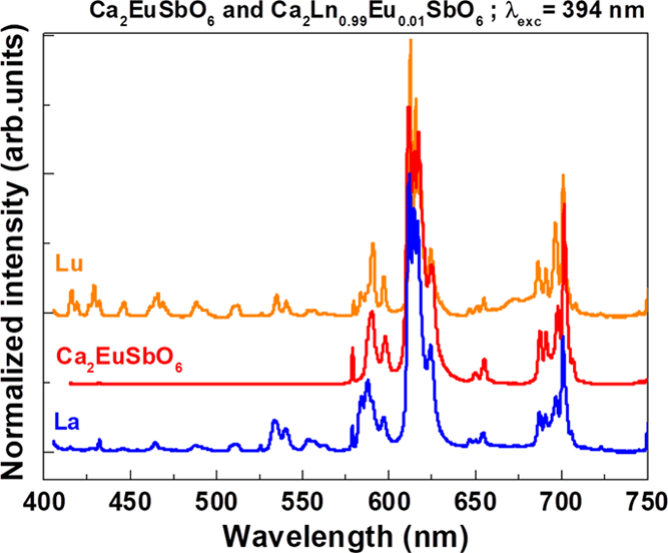
Room-temperature luminescence emission spectra of 1% Eu^3+^-doped Ca_2_LnSbO_6_ (Ln = Lu and La) and Ca_2_EuSbO_6_ upon excitation at 394 nm. Similar spectra of the 1% Eu^3+^-doped Ca_2_LnSbO_6_ (Ln = Y, Gd) are reported in Figure S3.

**Figure 5:**
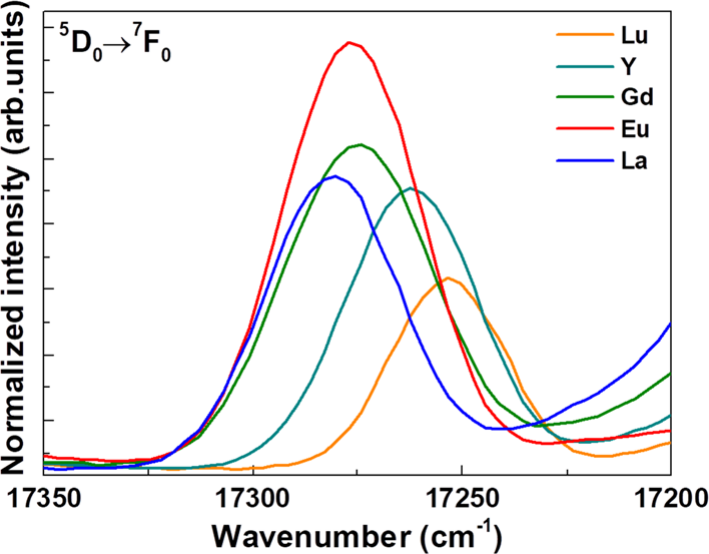
Details of the ^5^D_0_ → ^7^F_0_ emission band upon excitation at 394 nm.

**Figure 6:**
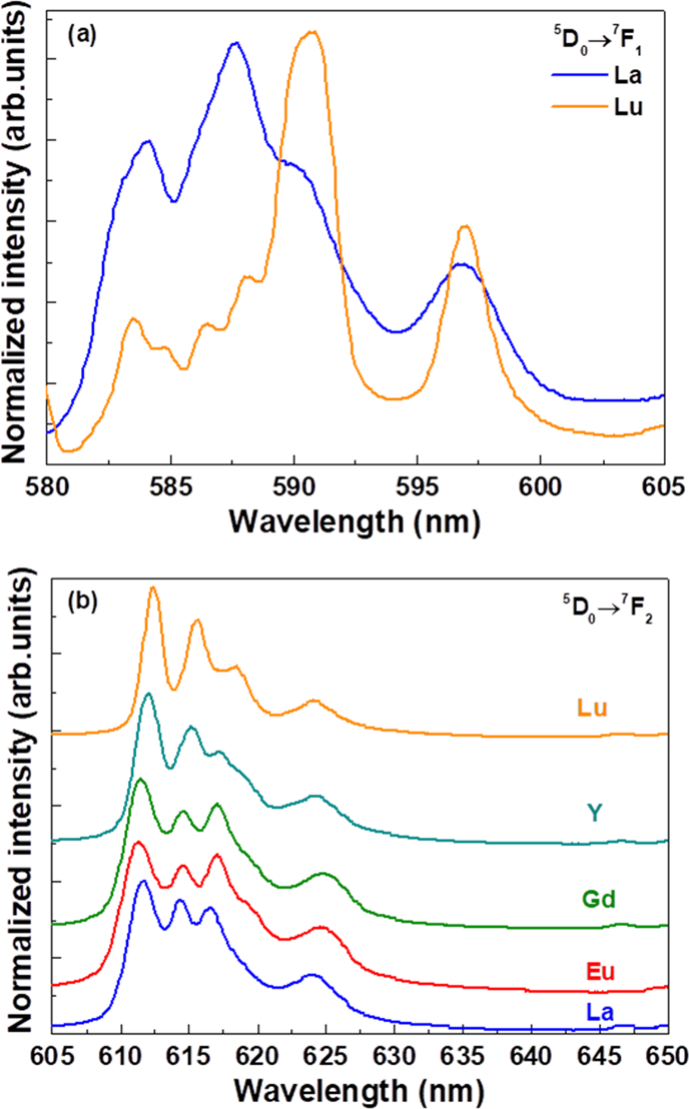
Emission upon 394 nm excitation: (a) ^5^D_0_ → ^7^F_1_ transition for 1% Eu^3+^-doped Ca_2_LaSbO_6_ and Ca_2_LuSbO_6_; (b) ^5^D_0_ → ^7^F_2_ emission band for 1% Eu^3+^-doped Ca_2_LnSbO_6_.

**Figure 7:**
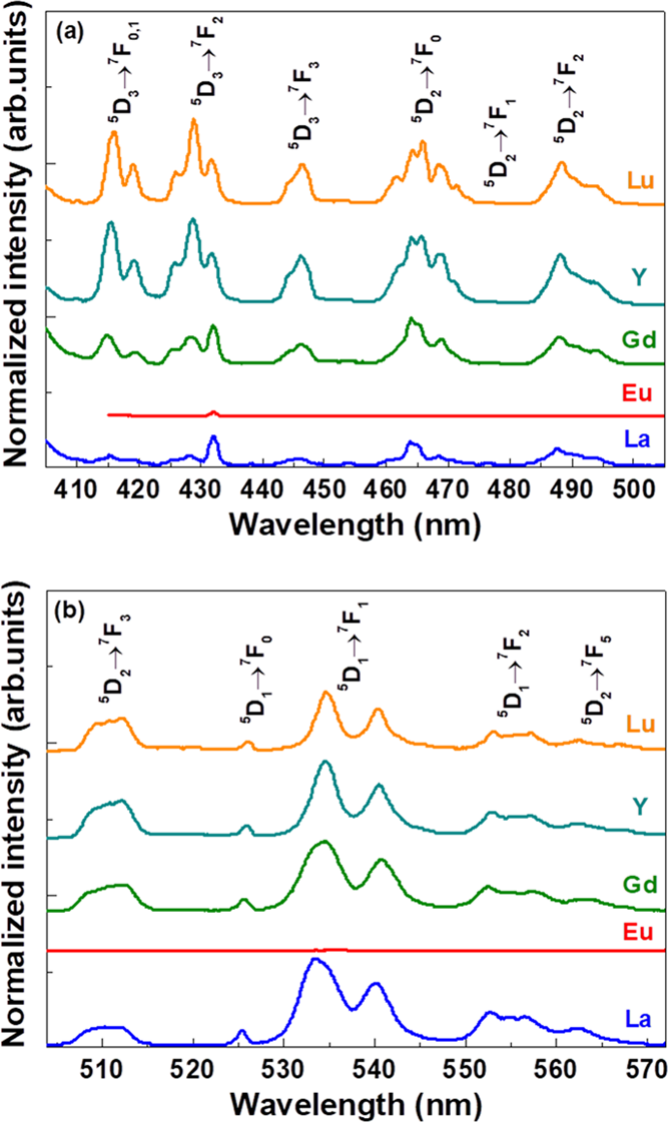
Details of the spectra of the 1% Eu^3+^-doped Ca_2_LnSbO_6_ and Ca_2_EuSbO_6_ phosphors upon excitation at 394 nm. (a) 400–500 nm range, bands originating in the ^5^D_3_ and ^5^D_2_ levels; (b) 500–570 nm range, bands originating in the ^5^D_2_ and ^5^D_1_ levels.

Upon excitation at 394 nm, we have obtained the luminescence emission spectra shown in Figure 4 (the Lu-, Eu-, and La-based compounds are chosen as representative samples). All the emission spectra (Figures 4–7) have been normalized to the *I*_max_ of the ^5^D_0_ → ^7^F_2_ transition.

The peaks above 570 nm are mainly assigned to emission bands originating from Eu^3+ 5^D_0_ excited state. A close inspection of the peak around 580 nm (17,241 cm^–1^, ^5^D_0_ → ^7^F_0_ transition) shows significant differences between the different compounds (Figure 5 and Table 4).

**Table 4:** Peak Position and Full Width Half Maximun (FWHM) of the ^5^D_0_ → ^7^F_0_ Transition in the Investigated Samples

host cation	peak position (nm)	peak position (cm^–1^)	FWHM (cm^–1^)
Lu	579.6	17,253	31.4
Y	579.3	17,262	36.2
Gd	578.9	17,275	42.4
Eu	578.8	17,276	39.5
La	578.6	17,281	38.9

We note that the 0-0 peak energies decrease along the lanthanide series (La → Lu). On the other hand, the full width half maximum (FWHM) is slightly larger for Ca_2_GdSbO_6_, Ca_2_EuSbO_6_, and Ca_2_LaSbO_6_ (close to 40 cm^–1^) than for Ca_2_YSbO_6_ and Ca_2_LuSbO_6_ hosts (36 and 31 cm^–1^, respectively). The Eu^3+^ ion, which is supposed to substitute for the Ln^3+^ one, can be located in the two available crystal sites (sites A and B, Table 2). In principle, due to the presence of only one Stark level both for ^5^D_0_ and ^7^F_0_, there should be a one-to-one correspondence between the number of the 0-0 emission bands and the number of emitting crystal sites. Seemingly, even though only one 0-0 component is detected for all materials, the broader peak could possibly be due to the presence of two overlapping 0-0 bands corresponding to two emitting Eu^3+^ crystal sites. This seems to be more evident in the case of Gd-, Eu-, and La-based compounds, where the 0-0 feature is broader. This statement is confirmed by the crystal chemistry for the following reasons: (i) the Eu^3+^ ion can occupy both available crystal sites in Ca_2_EuSbO_6_ (Table 2); (ii) in the case of Ca_2_LnSbO_6_ hosts (with Ln = Gd, Eu, and La), the similarity of the Ca(Ln)–O bond distances for each site suggests the presence of Eu in both available positions (Table 3). On the other hand, in light of its short Ca(Ln)–O distances (2.28 Å for Y and 2.18 Å for Lu, Table 3 and Figure S2), the crystal site B appears too small to accommodate Eu^3+^ in the case of Ca_2_YSbO_6_ and Ca_2_LuSbO_6_ matrices. In fact, the sum of the ionic radii of Eu and O (2.35 Å; CN = 6) is significantly higher than the aforementioned Ca(Ln)–O distances. In these hosts, the probable predominant occupation of site A by the Eu^3+^ ion is supported by the smaller FWHM of the 0-0 peak (Table 4). As far as the 0-0 peak position (transition energy) is concerned, it is well known that it can provide information about the covalency of the donor atom–Eu^3+^ bonds. This feature, which is strictly related to the nephelauxetic effect, would predict a decrease in the 0-0 transition energy upon an increase in the donor atom–Eu^3+^ bond covalency.^13,14^ Nevertheless, since the factors affecting the ^5^D_0_ energy of Eu^3+^ are many and still a subject of debate in the literature,^15^ we prefer, in the present contribution, to not discuss further this aspect. At this stage, it is useful to point out that the electric dipole transitions in the emission spectrum of Eu^3+^ located in the centrosymmetric crystal site B are highly forbidden and only the magnetic dipole-allowed ^5^D_0_ → ^7^F_1_ transition should be detectable. Nevertheless, in related antimonate materials, we demonstrated that the presence of cationic disorder (Ca and Ln, in the present case) induces the removal of the local inversion symmetry in the case of ions formally occupying centrosymmetric sites, from a crystallographic point of view.^16^ Therefore, also the Eu^3+^ emission from the cationic site B in Ca_2_LnSbO_6_ can occur through a forced electric dipole mechanism and, therefore, also the ^5^D_0_ → ^7^F*_J_* (*J* = 0, 2, 3, and 4) transition can be detected. The different luminescence emission features of Eu^3+^ [*i.e.*, ^5^D_0_ → ^7^F*_J_* (*J* = 1 and 2); Figure 6] are likely to be related to a different occupation of the crystal sites by Eu^3+^ along the Ca_2_LnSbO_6_ family.

In the case of Eu^3+^-doped Ca_2_LuSbO_6_ and Ca_2_YSbO_6_, the components of the emission manifolds are sharper and located at different values of wavelengths compared to the ones of the samples having Ca_2_GdSbO_6_ and Ca_2_LaSbO_6_ as hosts. Furthermore, the more complex emission pattern and the broader emission peaks are compatible with a multisite emission in the case of Ca_2_GdSbO_6_, Ca_2_EuSbO_6_, and Ca_2_LaSbO_6_. In particular, the emission profile of the ^5^D_0_ → ^7^F_2_ transition is reasonably similar in all the materials under investigation (Figure 6b); this agrees with the fact that for the 0-2 hypersensitive transition, only the noncentrosymmetric sites significantly contribute to the emission intensity. On the other hand, the behavior of the emission profile is much more complex in the case of the ^5^D_0_ → ^7^F_1_ magnetic dipole-allowed transition; as shown in Figure 6a, the 0-1 band has very different shapes for Ca_2_LaSbO_6_ and Ca_2_LuSbO_6_. This is due to the fact that in the former host (representative of a large Ln ion), Eu^3+^ is also located in centrosymmetric sites that give their contribution to the emission intensity, together with the noncentrosymmetric ones, but with different crystal field splitting, giving rise to broader features.

In the case of Eu^3+^-doped samples, and not of neat Ca_2_EuSbO_6_, luminescence from the high-energy levels ^5^D_3_, ^5^D_2_, and ^5^D_1_ has been also detected (Figure 7) upon excitation at 394 nm. *i.e.*, in the ^5^L_6_ level.^17^ This is made possible by the relatively low energy vibrational modes of the antimonate double perovskite host (ν∼ ≤ 800 cm^–1^)^18^ that makes multiphonon relaxation among the ^5^D*_J_* levels not fully efficient, given the values of the energy gaps between these levels (2549–2592 cm^–1^ for ^5^D_3_-^5^D_2_, 2511–2515 cm^–1^ for ^5^D_2_-^5^D_1_, and 1724–1750 cm^–1^ for ^5^D_1_-^5^D_0_). The situation is similar to the one reported many years ago for YVO_4_:Eu^3+^ (zircon phase),^19^ where the maximum phonon energy is around 900 cm^–1^,^20^ while in the case of the fluoride β-NaYF_4_ host, having dominant energy vibrational modes located between 300 and 400 cm^–1^,^21^ emission can be observed also from the ^5^L_6_ level and even higher-energy ones.^22^ This is not possible in oxide-based hosts. We also note that the ^5^D_1_-^5^D_0_ energy gap (∼1700 cm^–1^) cannot be efficiently bridged by multiphonon relaxation due to a selection rule that occurs if the two involved levels have *J* = 0 and *J* = 1.^23,24^

The observed behavior can be explained on the basis of cross-relaxation processes, leading to the depopulation of the high-energy ^5^D*_J_* (*J* = 1, 2, and 3) levels at high Eu^3+^ concentrations.^25^ These processes are identified as energy transfer mechanisms involving the ^7^F_0_ ground level or the thermally populated first excited level (^7^F_1_ and ^7^F_2_ above ^7^F_0_). The almost resonant transitions are:
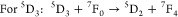

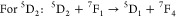

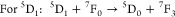


The mismatches are relatively small so that the cross-relaxation processes are presumably almost resonant. It is well known that the energy transfer probabilities significantly decrease as the intracenter distances increase; this agrees with a fast cascade depopulation of ^5^D_3_, ^5^D_2_, and ^5^D_1_ to ^5^D_0_ in fully concentrated Ca_2_EuSbO_6_. For this reason, only ^5^D_0_ is emissive in the neat material, where the shortest Eu^3+^–Eu^3+^ distance is equal to only 3.27(2) Å and energy transfer can be efficient.

**Figure 8:**
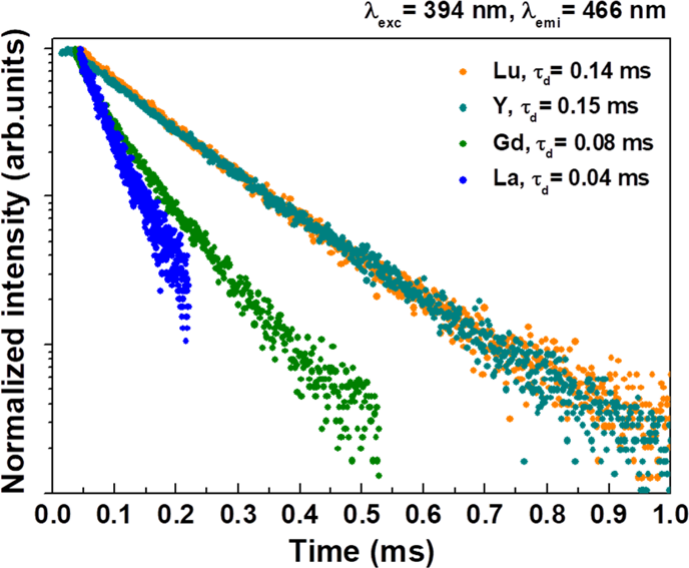
Decay curve of the ^5^D_2_ level upon 394 nm excitation for 1% Eu^3+^-doped Ca_2_LnSbO_6_.

**Figure 9:**
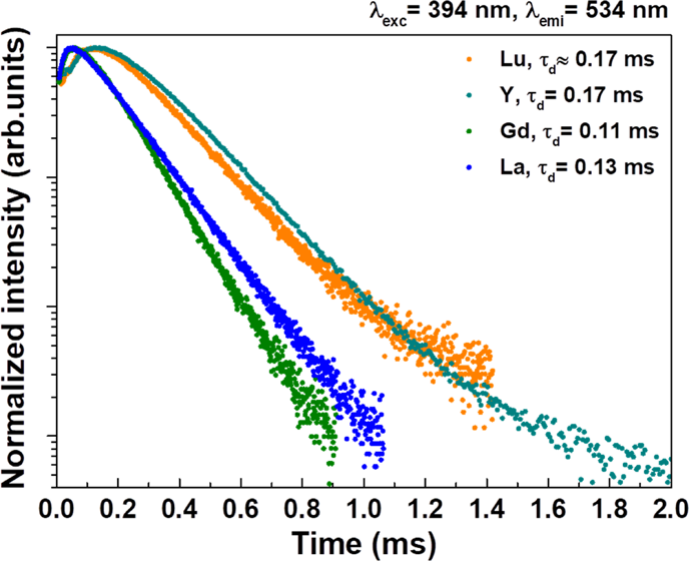
Decay curve of the ^5^D_1_ level upon 394 nm excitation for 1% Eu^3+^-doped Ca_2_LnSbO_6_.

**Figure 10:**
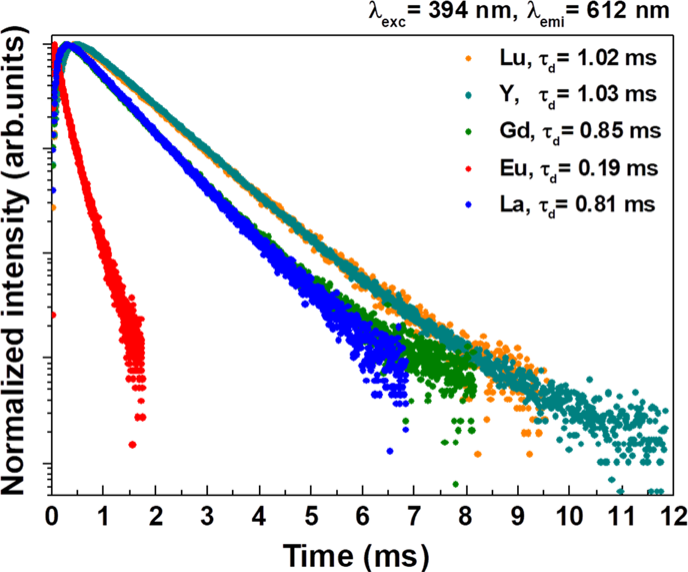
Decay curve of the ^5^D_0_ level upon 394 nm excitation for 1% Eu^3+^-doped Ca_2_LnSbO_6_.

The decay curves of the ^5^D_3_ level were found to be too fast to be measured with present equipment. On the other hand, the temporal evolution of the emission intensity after pulsed excitation at 394 nm was measured for the ^5^D_0_ level for all samples and for ^5^D_2_ and ^5^D_1_ for 1% Eu^3+^-doped Ca_2_LnSbO_6_ (Ln = Lu, Y, Gd, and La) (see Figures 8–10).

In the case of the ^5^D_2_ level, upon excitation at 394 nm, the decay curves for the doped samples show an extremely short buildup of the emission intensity, followed by a decay that is exponential for Ln = Lu and Y and non-exponential for Ln = Gd and La. The decay times are about 0.15 ms for the former materials and 0.04–0.08 ms for the latter (*e*-folding time), indicating that the cross-relaxation is more efficient for Ln = Gd and La (Figure 8).

As for the temporal evolution of the ^5^D_1_ emission, it is characterized by a clear rise that is clearly longer for Ln = Lu and Y than for Gd and La. The rise time cannot be properly evaluated but is in the region of tens of microseconds. This is followed by a nearly exponential decay with rates that are in the regions of 0.17 ms for Ln = Lu and Y and 0.11–0.13 ms for Gd and La (Figure 9).

Finally, in the case of the ^5^D_0_ level, upon pulsed excitation at 394 nm, a clear rise is observed for the doped materials due to feeding from the upper levels. This rise is approximately in the region of 0.10–0.30 ms, being longer for Ln = Lu and Y than for Ln = Gd and La. The decay is nearly exponential, with ^5^D_0_ lifetimes of about 1.02 ms for Ln = Lu and Y and 0.83 ms for Gd and La, although longer components seem to be present in the long time tail of the decay curve (Figure 9). As for Ca_2_EuSbO_6_, an exponential decay is observed, with a decay constant of 0.19 ms (Figure 10).

The short and exponential ^5^D_0_ decay in the neat Eu compound is clearly due to the presence of migration in this level in the Eu^3+^ subset of ions, until a killer center is reached and nonradiative relaxation occurs. This behavior has been reported many times in the literature for neat crystals containing Eu^3+^ (for instance, see Kellendonk and Blasse’s study^26^). As noted above, the absence of a buildup time clearly indicates that the population of ^5^D_0_ from the higher lying level is fast, as expected for cross-relaxation in the neat material.

On the other hand, the observed rise times of ^5^D_0_ and ^5^D_1_ appear to be close to the decay times of the level lying immediately above. This is compatible with their sequential population from the level above through cross-relaxation, in agreement with the rate equation model proposed by Berdowski and Blasse and for Eu^3+^ in NaGdTiO_4_.^27^

In general, the results obtained upon pulsed excitation appear to be different for the doped samples with Ln = Lu and Y with respect to the ones with Ln = Gd and La. In parallel, the emission intensity from the ^5^D_3_ level is significantly lower for Gd- and La-based hosts (Figure 7a). This peculiar behavior can find a tentative explanation if we consider a more efficient energy transfer mechanism by cross-relaxation between Eu^3+^ ions, where the Eu–Eu distances are, on average, shorter. In this context, the ^5^D_3_ level is more efficiently depopulated and the lifetimes of ^5^D_0_, ^5^D_1_, and ^5^D_2_ levels are shorter. This is the case of Ca_2_LaSbO_6_ and Ca_2_GdSbO_6_ hosts, in which both available A and B crystal sites are supposed to be occupied by Eu^3+^ and the shortest Eu–Eu distance is around 3.29(1) Å (in Ca_2_GdSbO_6_), corresponding to the smallest distance between sites A and B. Otherwise, in Ca_2_LuSbO_6_, where the luminescent ion is supposed to selectively occupy site A, the shortest possible Eu–Eu distance is larger than 3.29 Å [3.90(1) Å (the distance between two adjacent sites A)]. Nevertheless, in view of the complex crystal structure of the hosts and the structural disorder possibly inducing the breaking of the local inversion symmetry of B sites,^16^ other factors can contribute to determine the observed spectroscopic features.

**Figure 11:**
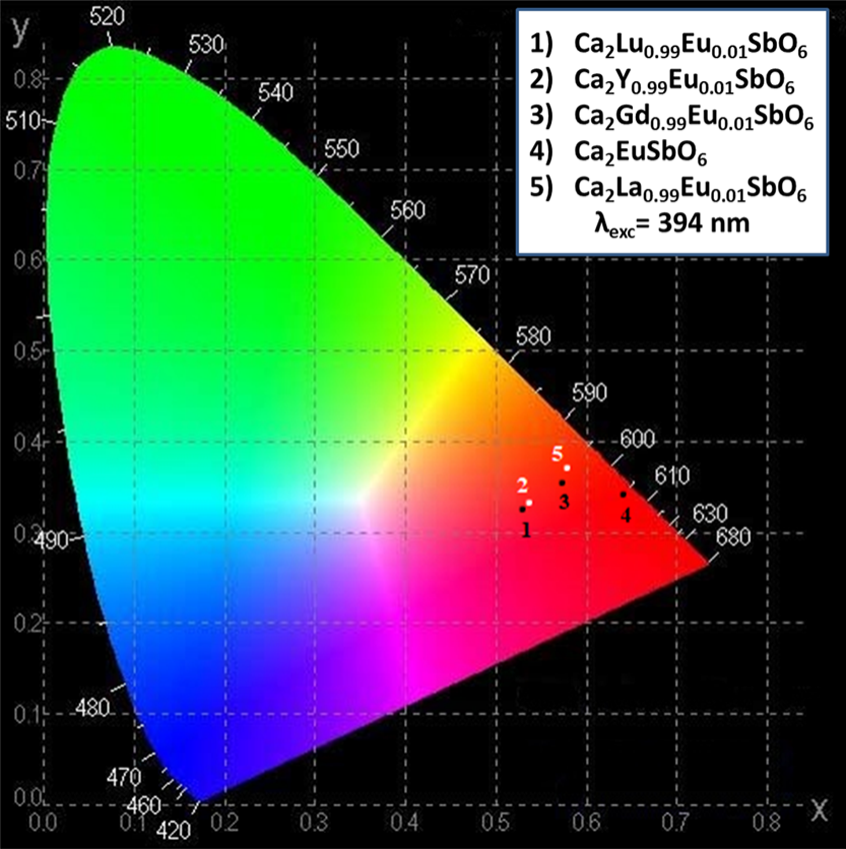
CIE coordinate diagram of 1% Eu^3+^-doped Ca_2_LnSbO_6_ upon excitation at 394 nm.

It is interesting to note that due to the different contributions of the emission stemming from ^5^D_3_, ^5^D_2_, ^5^D_1_ (in the blue and green spectral regions), and ^5^D_0_ (mainly in the red), the final emission color can be tuned. The point 4 in the CIE diagram (Figure 11) (corresponding to an almost pure red color in Ca_2_EuSbO_6_) can be moved toward the green region (points 3 and 5) thanks to the presence of a significant green component (the ^5^D_1_ → ^7^F_1_ band around 535 nm) in Ca_2_GdSbO_6_ and Ca_2_LaSbO_6_ hosts. A significant blue component (410–430 nm, corresponding to the ^5^D_3_ → ^7^F_1,2_ transitions) has been observed for the Ca_2_LuSbO_6_ and Ca_2_YSbO_6_ hosts so that points 1 and 2 are closer to the blue spectral region (Figure 11).

## 4 Conclusions

Eu^3+^ (1 mol %)-doped Ca_2_LnSbO_6_ (Ln = Lu, Y, Gd, and La) samples show unusual spectroscopic features, which are connected to the low-energy vibrational modes of the antimonate double perovskite and to the different site distribution of the luminescent Eu^3+^ dopant ions. In particular, the color of the emitted light can be tuned by simply acting on the nature of Ln ions in the host. The typical red emission of Ca_2_EuSbO_6_ can be slightly shifted toward the green and blue spectral regions when 99% of Eu is replaced by La (or Gd) and by Lu (or Y), respectively. This is made possible thanks to the inefficient multiphonon relaxation process among ^5^D*_J_* levels in the doped samples. In this way, we observe emission in the 460–570 nm region (from ^5^D_1_ and ^5^D_2_) in all the Eu^3+^-doped samples and emission in the 400–450 nm region (from the ^5^D_3_ level) mainly in the Eu^3+^-doped Ca_2_LuSbO_6_ and Ca_2_YSbO_6_ materials. In general, the emission from the ^5^D*_J_* (*J* = 0–3) levels of Eu^3+^ is more efficient in the Y- and Lu-based hosts and a possible explanation for this behavior can be found in the crystal chemistry of these materials. The structural investigation on the host matrix suggests a different occupation of the cationic sites by the Eu^3+^ dopant, which should be preferentially located in the A site when Ln = Y or Lu, while they can occupy both available crystal sites (A and B) in the case of Ca_2_LaSbO_6_ and Ca_2_GdSbO_6_ hosts.

Although the cross-relaxation mechanism involving Eu^3+^ ions (a nonradiative energy transfer pathway) is weakly active in all the doped samples, it is expected to be more efficient when the Eu–Eu distances are shorter. This is the case of the La- and Gd-based compounds, where the shortest possible Eu–Eu distance is around 3.29 Å *vs* 3.90 Å in the case of Ca_2_LuSbO_6_. As a consequence, the emission intensity from the ^5^D_3_ level is significantly lower and the decay of the ^5^D_0_, ^5^D_1_, and ^5^D_2_ levels is faster for Gd- and La-based hosts. Finally, as a consequence of the drastic increase in Eu^3+^ concentration, in the case of Ca_2_EuSbO_6_, an efficient cross-relaxation mechanism involving ^5^D*_J_* levels takes place and no emission from ^5^D*_J_* levels (*J* = 1, 2, and 3) is possible. In addition, a fast migration of the energy toward killer centers is responsible for a drastic decrease in ^5^D_0_ excited state lifetime.

This contribution clearly shows how important is the detailed knowledge of the host crystal chemistry and of the nonradiative mechanisms taking place to control the luminescence features of an optical material.

## Supporting Information Available

Lists of crystal data and luminescence emission spectra for the materials under investigation (PDF)
