# Supplementary material for: Systematic Analysis of the Crystal Chemistry and Eu3+ Spectroscopy along the Series of Double Perovskites Ca2LnSbO6 (Ln = La, Eu, Gd, Lu, and Y)
Source: Inorg Chem. 2021 May 21;60(11):8259–66. doi: 10.1021/acs.inorgchem.1c00932 (PMC8277163; doi:10.1021/acs.inorgchem.1c00932)

Systematic analysis of the crystal chemistry and the Eu^{3+} spectroscopy along the series of the double perovskite Ca_2LnSbO_6 (Ln = La, Eu, Gd, Lu and Y)

Fabio Piccinelli*[,1], Irene Carrasco[2], Chong-Geng Ma[3], and Marco Bettinelli*[,1]

[1] Luminescent Materials Laboratory, DB, Università di Verona, and INSTM, UdR Verona, Strada Le Grazie 15, 37134 Verona, Italy

2 Univ. Rennes, CNRS, ISCR – UMR 6226, ScanMAT – UMS 2001, IETR – UMR 6164, Département Microélectronique & Microcapteurs, F-35000 Rennes, France

[3] College of Science, Chongqing University of Posts and Telecommunications, Chongqing, 400065, PR China

* Corresponding authors. Email addresses: fabio.piccinelli@univr.it; marco.bettinelli@univr.it.

Electronic Supporting Information

Table S1. Crystal data for Ca_2GdSbO_6:1%Eu^{3+}

Atom type	Site multiplicity	Fractional atomic coordinates			Occupation Factors (OF's)	U_{iso}
		x/a	y/b	z/c		
Sb	*2a*	0	0	0	1	0.009(5)
Ca1/Gd1	*2b*	0	0	0.5	0.750(5)/0.250(5)	0.018(2)
Ca2/Gd2	*4e*	0.4986(5)	0.9410(2)	0.2525(3)	0.625(6)/0.375(6)	0.010(4)
O1	*4e*	0.133 (3)	0.067 (3)	0.218(3)	1	0.023(6)
O2	*4e*	0.687(3)	0.150(2)	0.056(2)	1	0.042(4)
O3	*4e*	0.178(3)	0.293(3)	0.953(2)	1	0.038(5)

Table S2. Crystal data for Ca_2LaSbO_6:1%Eu^{3+}

Atom type	Site multiplicity	Fractional atomic coordinates			Occupation Factors (OF's)	U_{iso}
		x/a	y/b	z/c		
Sb	*2a*	0	0	0	1	0.012(5)
Ca1/La1	*2b*	0	0	0.5	0.980(2)/0.020(2)	0.015(2)
Ca2/La2	*4e*	0.5125(4)	0.9488(2)	0.2497(2)	0.510(2)/0.490(2)	0.011(2)
O1	*4e*	0.104(2)	0.057(1)	0.238(1)	1	0.031(6)
O2	*4e*	0.705(1)	0.174(1)	0.047(1)	1	0.040(6)
O3	*4e*	0.157(2)	0.286(2)	0.938(2)	1	0.035(3)

Table S3. Crystal data for Ca_2LuSbO_6:1%Eu^{3+}

Atom type	Site multiplicity	Fractional atomic coordinates			Occupation Factors (OF's)	U_{iso}
		x/a	y/b	z/c		
Sb	*2a*	0	0	0	1	0.010(4)
Ca1/Lu1	*2b*	0	0	0.5	0.064(3)/0.936(3)	0.016(3)
Ca2/Lu2	*4e*	0.5157(8)	0.9466(4)	0.2479(7)	0.968(2)/0.032(2)	0.010(2)
O1	*4e*	0.123(1)	0.051(2)	0.236(2)	1	0.037(5)
O2	*4e*	0.703(2)	0.187(2)	0.054(3)	1	0.044(7)
O3	*4e*	0.187(2)	0.296(2)	0.939(2)	1	0.031(4)

Table S4. Crystal data for Ca_2YSbO_6:1%Eu^{3+}

Atom type	Site multiplicity	Fractional atomic coordinates			Occupation Factors (OF's)	U_{iso}
		x/a	y/b	z/c		
Sb	*2a*	0	0	0	1	0.008(4)
Ca1/Y1	*2b*	0	0	0.5	0.128(2)/0.872(2)	0.011(4)

Ca2/Y2	*4e*	0.5159(5)	0.9445(2)	0.2457(4)	0.870(2)/0.130(2)	0.010(3)
O1	*4e*	0.120(1)	0.060(1)	0.229(1)	1	0.031(3)
O2	*4e*	0.718(1)	0.188(1)	0.052(1)	1	0.039(6)
O3	*4e*	0.170(1)	0.274(1)	0.933(1)	1	0.035(4)

Table S5. Crystal data for Ca_2EuSbO_6

Atom type	Site multiplicity	Fractional atomic coordinates			Occupation Factors (OF's)	U_{iso}
		x/a	y/b	z/c		
Sb	*2a*	0	0	0	1	0.011(5)
Ca1/Eu1	*2b*	0	0	0.5	0.829(2)/0.171(2)	0.021(3)
Ca2/Eu2	*4e*	0.5163(3)	0.9409(2)	0.2466(2)	0.586(3)/0.414(3)	0.012(4)
O1	*4e*	0.124(1)	0.053(1)	0.2288(9)	1	0.033(7)
O2	*4e*	0.712(1)	0.166(1)	0.064(1)	1	0.042(6)
O3	*4e*	0.167(1)	0.292(1)	0.922(1)	1	0.028(4)

Table S6. Indicators of the quality of the refinements of the samples under investigation

**Sample**	**Reliability factors**	
	**wRp(%)**	**Rp(%)**
Ca_2GdSbO_6:1%Eu^{3+}	11.39	8.72
Ca_2LaSbO_6:1%Eu^{3+}	11.68	9.19
Ca_2LuSbO_6:1%Eu^{3+}	8.16	6.53
Ca_2YSbO_6:1%Eu^{3+}	11.17	8.79
Ca_2EuSbO_6	8.52	6.94

(a)

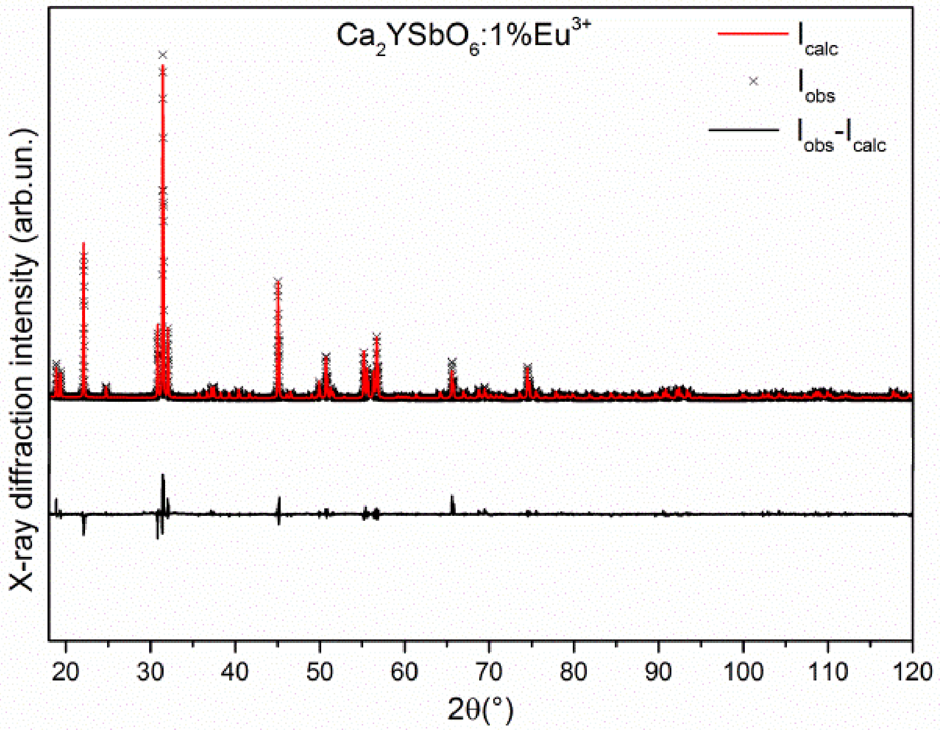

Ca2YSbO6:1%Eu3+
Icalc
Iobs
Iobs-Icalc
X-ray diffraction intensity (arb.un.)
20
30
40
50
60
70
80
90
100
110
120
2θ(°)

(b)

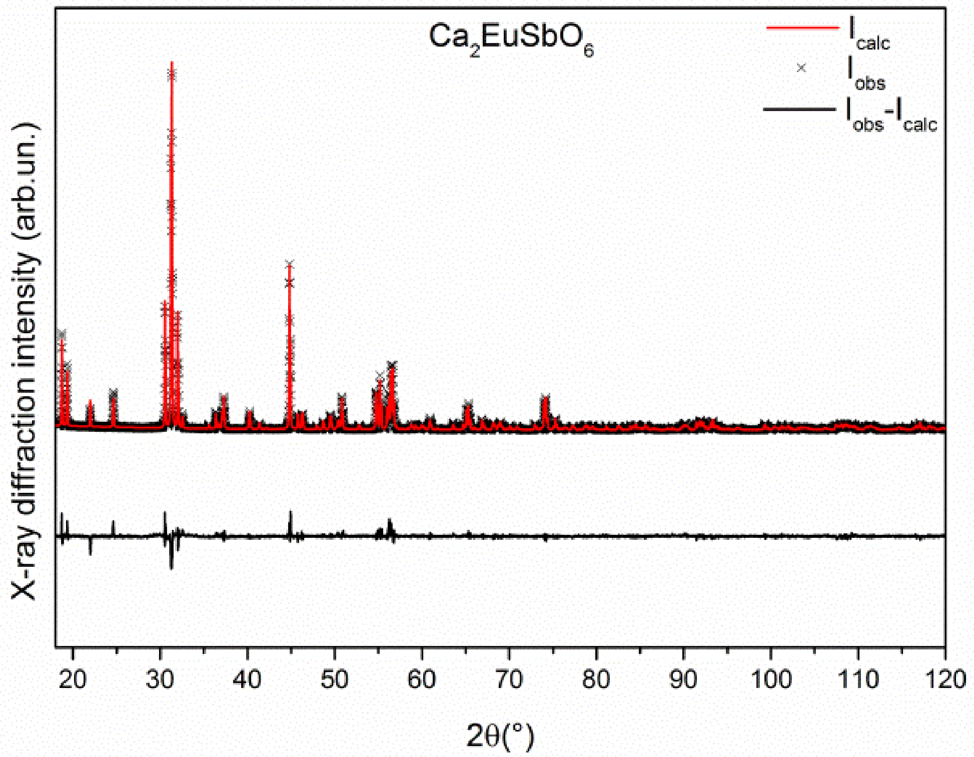

Ca2EuSbO6
Icalc
Iobs
Iobs-Icalc
X-ray diffraction intensity (arb.un.)
20
30
40
50
60
70
80
90
100
110
120
2θ(°)

Figure S1. Observed (crosses) and refined (continuous red line) powder patterns of (a) 1% Eu^{3+} doped Ca_2YSbO_6 and (b) Ca_2EuSbO_6. The observed-refined curves are shown at the bottom of each plot.

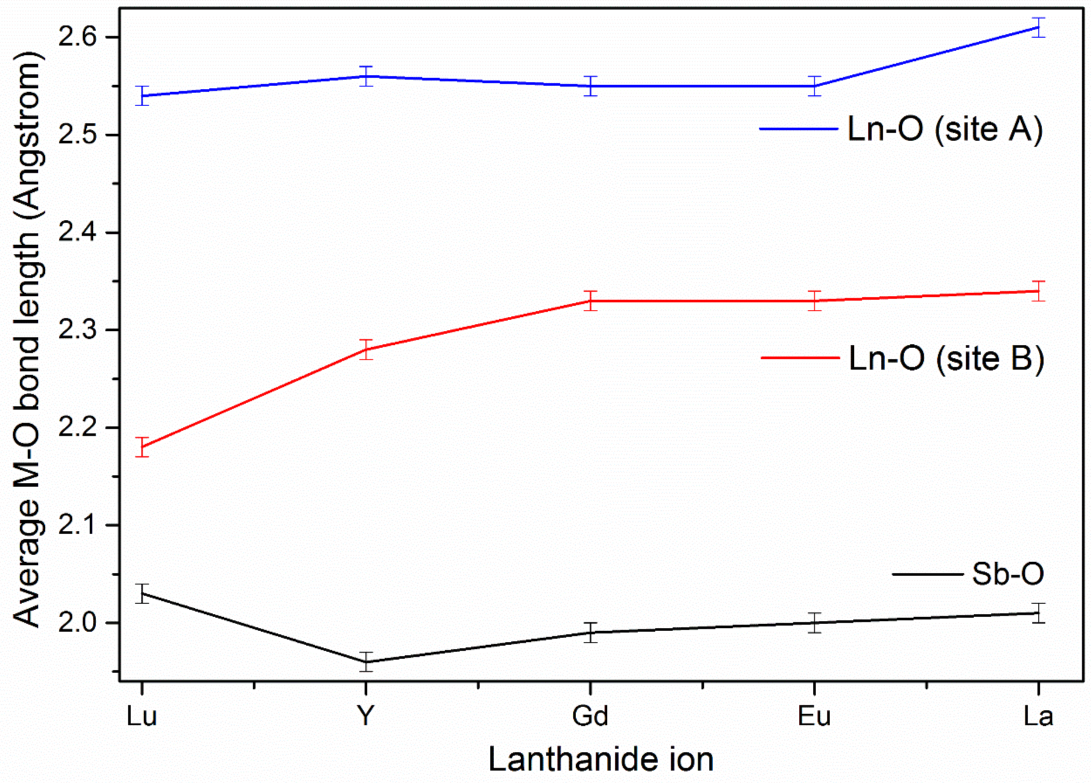

Figure S2. Plot of the M-O bond distances as a function the nature of the lanthanide ion in the Ca_2LnSbO_6 family

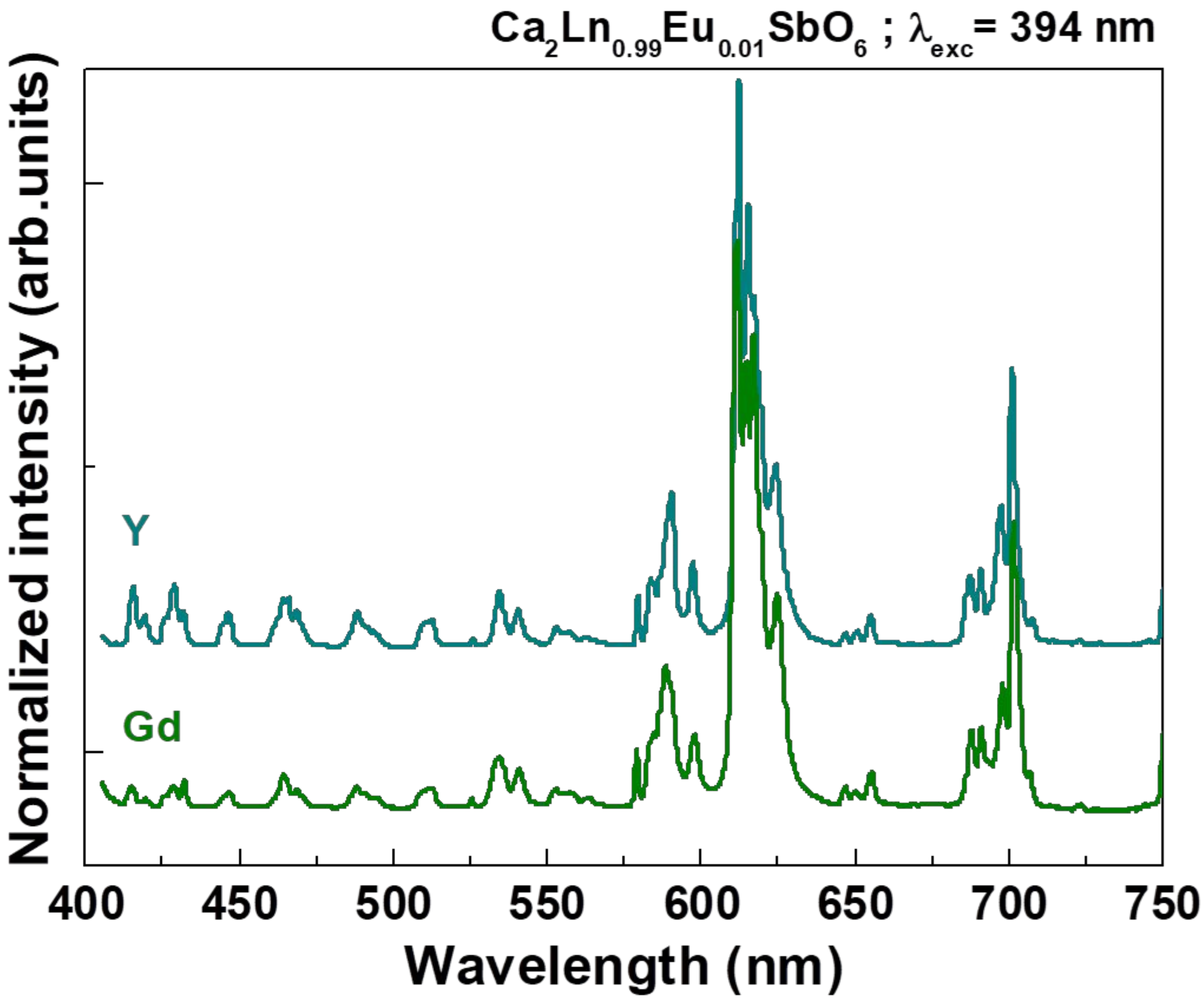

Figure S3. Room temperature luminescence emission spectra of 1% Eu^{3+} doped Ca_2LnSbO_6 (Ln = Y, Gd) upon excitation at 394 nm.

Powder Diffraction Data

Source: laboratory X-ray (λ =1.5418 Å)

Chemical formula: **$Ca_2La_{0.99}Eu_{0.01}SbO_6$**

Formula weight: 436.95 u.m.a.

Temperature: 298 K

Crystal system: Monoclinic

Space group: *$P2_1/n$*, No. 14

a(Å), b(Å), c(Å), β(°): 5.6830(1), 5.8795(1), 8.1707(1), 89.913(4)

V (Å^3): 273.01(2)

Z: 2

d-space range: 0.890-4.928 Å

χ^2 $(R_{wp}/R_{exp})^2$: 1.115

R_p $(\Sigma|y_o^i-y_c^i|/\Sigma y_o^i)$: 0.0919

R_{wp} (id., weighted): 0.1168

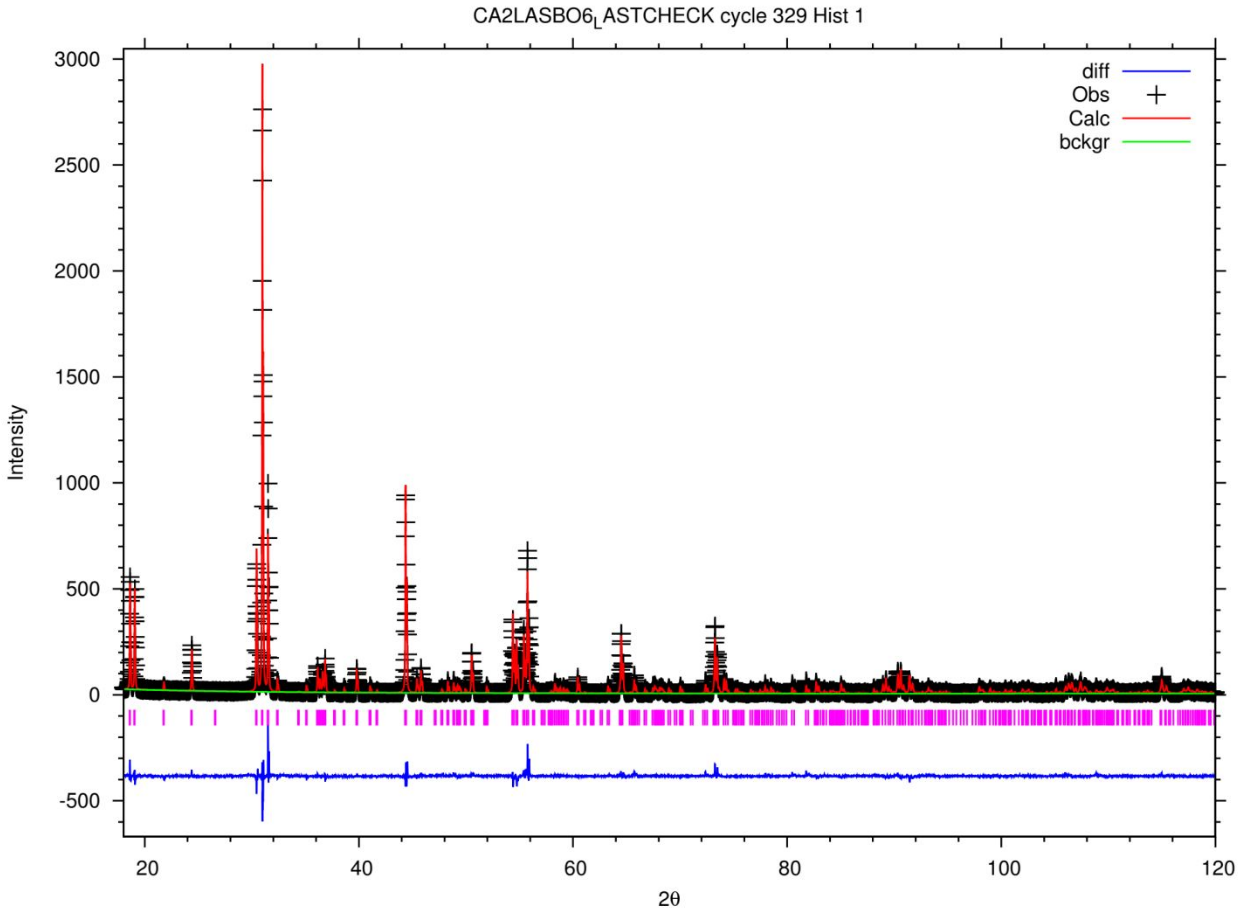

Chemical formula: **Ca_2EuSbO_6**

Formula weight: 449.88 u.m.a.

Temperature: 298 K

Crystal system: Monoclinic

Space group: *$P2_1/n$*, No. 14

a(Å), b(Å), c(Å), β(°): 5.5947(2), 5.8522(2), 8.0887(2), 90.255(3)

V (Å^3): 264.83(2)

Z: 2

d-space range: 0.890-4.928 Å

χ^2 $(R_{wp}/R_{exp})^2$: 1.204

R_p ($\Sigma|y_o^i-y_c^i|/\Sigma y_o^i$): 0.0694

R_{wp} (id., weighted): 0.0852

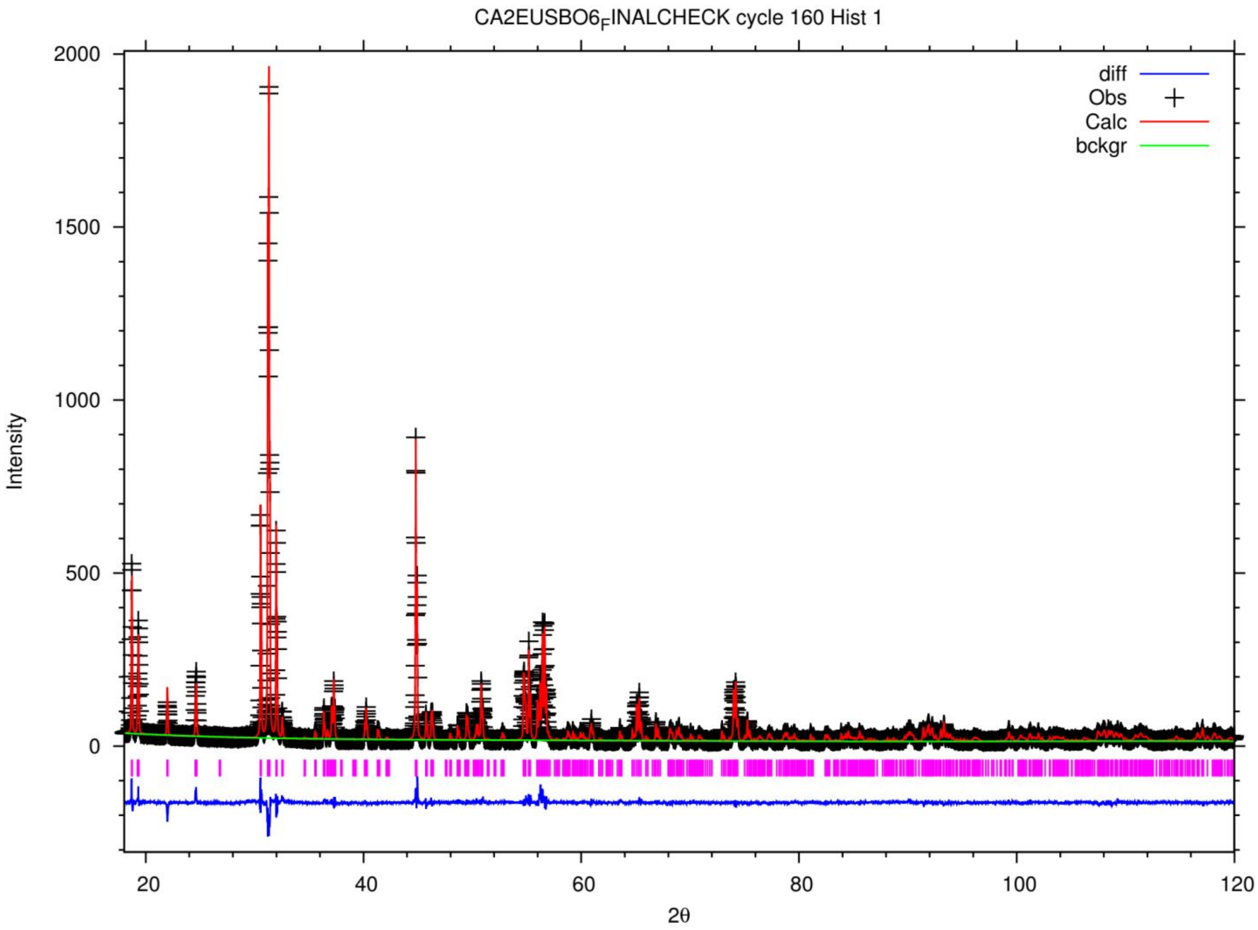

Chemical formula: **$Ca_2Gd_{0.99}Eu_{0.01}SbO_6$**

Formula weight: 455.11 u.m.a.

Temperature: 298 K

Crystal system: Monoclinic

Space group: *$P2_1/n$*, No. 14

a(Å), b(Å), c(Å), β(°): 5.5884(2), 5.8466(3), 8.0817(1), 89.753(5)

V (Å^3): 264.06(3)

Z: 2

d-space range: 0.890-4.928 Å

χ^2 $(R_{wp}/R_{exp})^2$: 1.360

R_p ($\Sigma|y_o^i-y_c^i|/\Sigma y_o^i$): 0.0872

R_{wp} (id., weighted): 0.1139

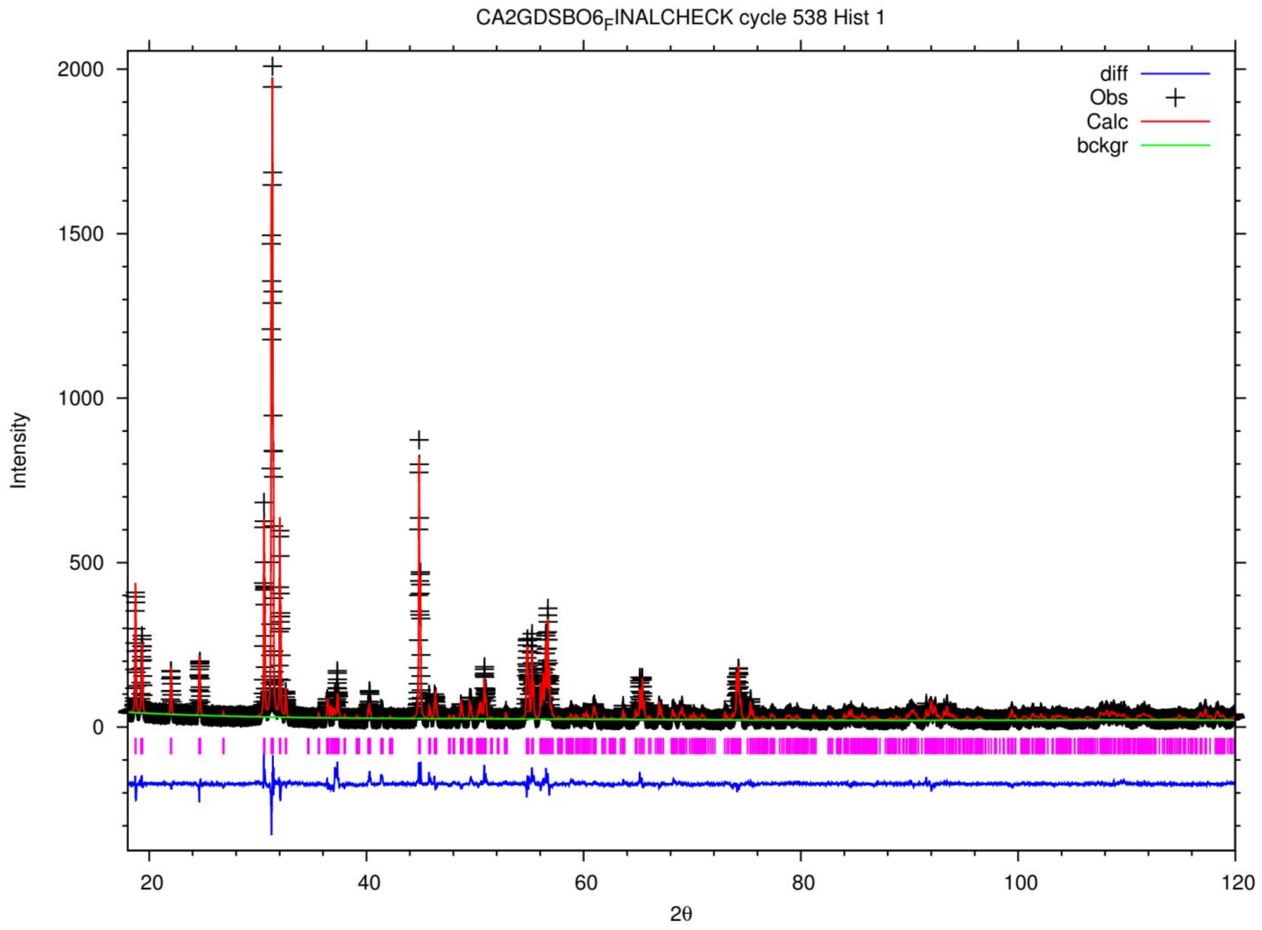

Chemical formula: **$Ca_2Y_{0.99}Eu_{0.01}SbO_6$**

Formula weight: 387.45 u.m.a.

Temperature: 298 K

Crystal system: Monoclinic

Space group: *P2₁/n*, No. 14

a(Å), b(Å), c(Å), β(°): 5.5888(1), 5.8021(1), 8.0494(3), 89.970(4)

V ($Å^3$): 261.01(1)

Z: 2

d-space range: 0.890-4.928 Å

χ^2 $(R_{wp}/R_{exp})^2$: 1.143

R_p $(\Sigma|y_o^i-y_c^i|/\Sigma y_o^i)$: 0.0879

R_{wp} (id., weighted): 0.1117

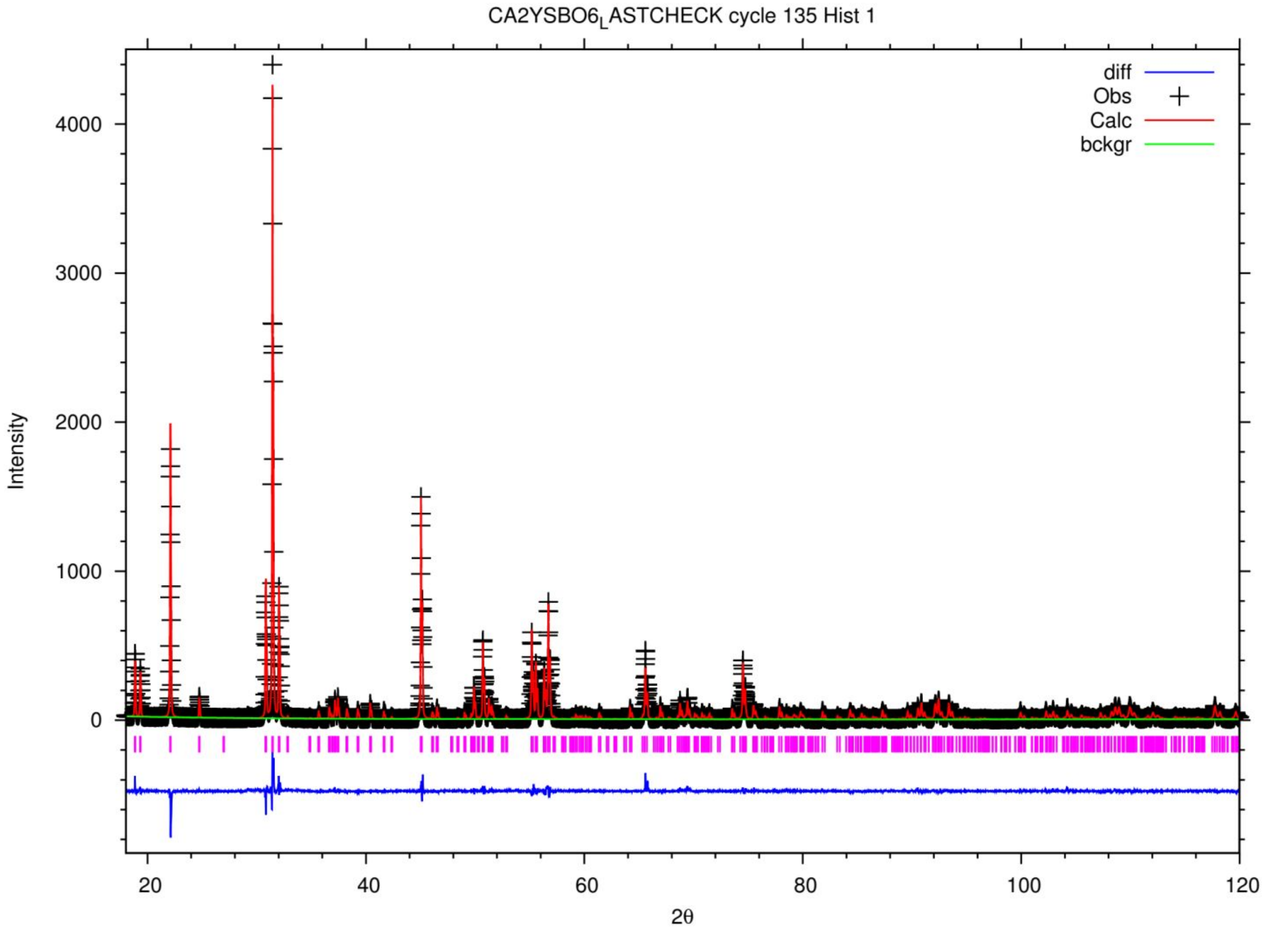

Chemical formula: $\mathbf{Ca_2Lu_{0.99}Eu_{0.01}SbO_6}$

Formula weight: 472.65 u.m.a.

Temperature: 298 K

Crystal system: Monoclinic

Space group: *P2₁/n*, No. 14

a(Å), b(Å), c(Å), β(°): 5.5711(1), 5.7530(1), 7.9958(3), 89.913(2)

V (Å^3): 253.27(2)

Z: 2

d-space range: 0.890-4.928 Å

χ^2 $(R_{wp}/R_{exp})^2$: 1.296

R_p $(\Sigma | y_o^i - y_c^i | / \Sigma y_o^i)$: 0.0653

R_{wp} (id., weighted): 0.0816

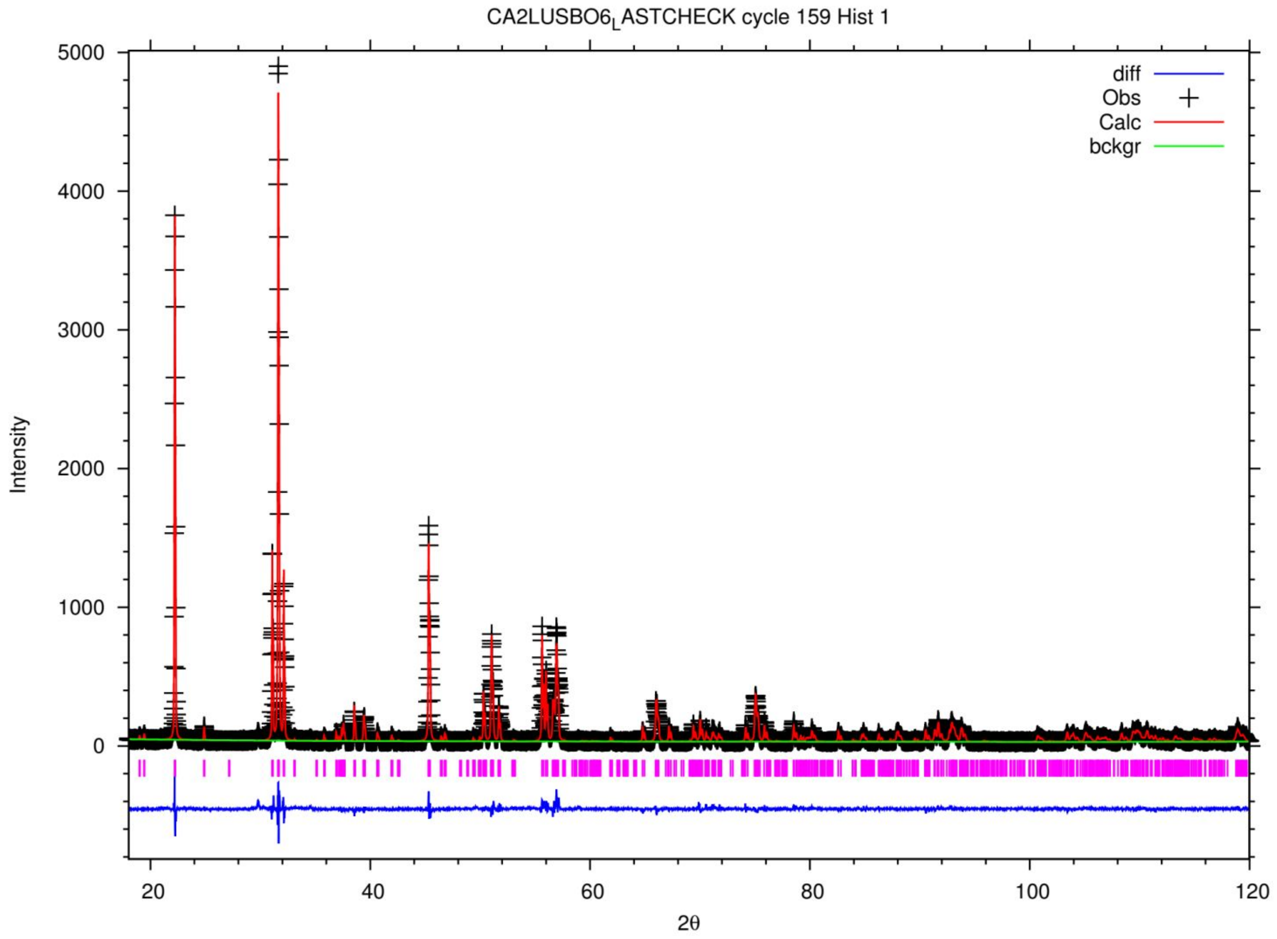

Supplement: Supplementary file 1 — ic1c00932_si_001.pdf [file ic1c00932_si_001.pdf]
